# Shape-Controlled Synthesis of Copper Indium Sulfide Nanostructures: Flowers, Platelets and Spheres

**DOI:** 10.3390/nano9121779

**Published:** 2019-12-14

**Authors:** Jiajia Ning, Stephen V. Kershaw, Andrey L. Rogach

**Affiliations:** Department of Materials Science and Engineering, and Centre for Functional Photonics (CFP), City University of Hong Kong, 83 Tat Chee Avenue, Kowloon, Hong Kong, China; jiajning@cityu.edu.hk (J.N.); skershaw@cityu.edu.hk (S.V.K.)

**Keywords:** CuInS_2_, nanospheres, nanoflowers, nanoplatelets, *tert*-dodecanethiol

## Abstract

Colloidal semiconductor nanostructures have been widely investigated for several applications, which rely not only on their size but also on shape control. CuInS_2_ (often abbreviated as CIS) nanostructures have been considered as candidates for solar energy conversion. In this work, three-dimensional (3D) colloidal CIS nanoflowers and nanospheres and two-dimensional (2D) nanoplatelets were selectively synthesized by changing the amount of a sulfur precursor (*tert*-dodecanethiol) serving both as a sulfur source and as a co-ligand. Monodisperse CIS nanoflowers (~15 nm) were formed via the aggregation of smaller CIS nanoparticles when the amount of *tert*-dodecanethiol used in reaction was low enough, which changed towards the formation of larger (70 nm) CIS nanospheres when it significantly increased. Both of these structures crystallized in a chalcopyrite CIS phase. Using an intermediate amount of *tert*-dodecanethiol, 2D nanoplatelets were obtained, 90 nm in length, 25 nm in width and the thickness of a few nanometers along the *a*-axis of the wurtzite CIS phase. Based on a series of experiments which employed mixtures of *tert*-dodecanethiol and 1-dodecanethiol, a ligand-controlled mechanism is proposed to explain the manifold range of the resulting shapes and crystal phases of CIS nanostructures.

## 1. Introduction

Colloidal semiconductor nanostructures have been extensively investigated due to their size dependent electronic structure and their attractive optical properties [1,2]. The band gap of semiconductor nanocrystals (NCs) can be conveniently tuned by changing the size, when it is comparable to or smaller than the Bohr exciton radius [3]. Beside the size, the shape also has a strong influence on the electronic structure and optical properties of semiconductor nanostructures [4,5]. For example, polarized emission is a characteristic property of one-dimensional (1D) semiconductor nanorods [6], which can be used for application in displays [7]. Two-dimensional (2D) semiconductor nanoplatelets (NPLs) have also been widely developed and investigated [5,8]. CdSe NPLs with a low number of layers, defined with atomic precision, show very narrow absorption and fluorescence spectra [7], and polarized emission was observed as well [9]. Size- and shape-controlled syntheses of Cd-based semiconductor nanostructures has been, in general, well developed: dot-, rod-, wire-, platelet-, tetrapod- or octapod-shaped NCs have all been produced and extensively studied [10,11,12]. However, the presence of the heavy metal Cd in these materials severely limits their applications in many commercial types of optoelectronic device and in biomedicine.

CuInS_2_ (often abbreviated as CIS) is a heavy metal-free and earth abundant ternary semiconductor, which has a direct band gap of ~1.5 eV and a Bohr exciton radius of ~4.1 nm [13]. It is a stable compound with a large extinction coefficient of ~10^5^ cm^−1^ at 500 nm, [14] which makes it one of the best materials for solar energy conversion. Thin film CIS solar cells have been widely developed, employing fabrication via sputtering or evaporation methods. [15] Colloidal synthesis is yet another popular approach towards the fabrication of CIS-based solar energy conversion devices. [16] Colloidal CIS NCs can be synthesized through the reaction of copper and indium salts (chlorides, iodides and carboxylates) with various sulfur precursors (thiourea, n-alkylthiols, and elemental sulfur), as well as by the decomposition of single molecule precursors [17]. Because of the higher reactivity of copper precursors, Cu_2_S NCs can form at the initial stage, and ternary CIS NCs can then be formed via a partial cation exchange. [18] Based on the cation exchange reactions, mixed-phase materials, such as Cu_2_S-In_2_S_3_ and Cu_2_S-CIS, were also reported in some cases [19,20]. We note that the majority of colloidal CIS nanostructures were mostly reported in dot- and rod-shapes [17], and broader tuning of the shapes is still a challenge. Herein, we have developed a colloidal method to synthesize CIS nanostructures with different sizes, shapes and crystal phases, ranging from small nanoflowers and larger-sized nanospheres with chalcopyrite crystal structure, and NPLs in the wurtzite phase. The shape and size control has been enabled by the use of *tert*-dodecanethiol (*t*-DDT) which played a crucial role in the formation of CIS nanostructures reported here.

## 2. Experimental Section

**Chemicals:** Indium (III) acetate (99.99%), copper (I) iodide (98%), *tert*-dodecanethiol (*t*-DDT, 98.5%), 1-dodecanethiol (1-DDT, 98.0%), 1-octadecene (ODE, 90%), toluene (99%, anhydrous), and ethanol (99.8%, anhydrous) were purchased from Sigma-Aldrich. Oleylamine (OLA, approximate C18 content 80−90%) was purchased from Acros.

The experiments below were carried out using standard airless techniques: a vacuum/dry Ar gas Schleck line was used for the syntheses.

**Synthesis of CIS nanoflowers:** Indium acetate (146.0 mg, 0.5 mmol), copper iodide (95.3 mg, 0.5 mmol), OLA (4.0 mL) and ODE (8.0 mL) were added into a three-neck flask which was then connected to a Schleck line. The mixture in the three-neck flask was degassed and heated to 90 °C, and the mixed solution was kept at 90 °C under vacuum for 1 h to remove any water from the system. Then the solution was heated to 180 °C to form a clear solution. At 180 °C, 0.5 mL of *t*-DDT was injected into the three neck flask. Then the reaction was kept at 180 °C for 1h. The crude CIS nanoflowers product solution was dissolved in toluene, and the CIS nanoflowers were precipitated by adding ethanol and with the aid of centrifugation. All of the CIS nanoflower products were purified by dissolving in toluene and precipitating with ethanol for three cycles, and were finally re-dissolved in toluene.

**Synthesis of CIS NPLs:** The synthetic process was similar to the synthesis of CIS nanoflowers, with the difference being that the injected amount of *t*-DDT was increased. Thus, 1.0 mL of *t*-DDT was injected into the three-neck flask at 180 °C with 0.5 mmol of copper precursor and 0.5mmol indium precursor in OLA and ODE. Then, the reaction was kept at 180 °C for 1 h. The crude CIS NPL product solution was dissolved in toluene, and the CIS NPLs, as above, were precipitated by adding ethanol with the aid of centrifugation. Again, all of the CIS NPL products were purified by dissolving in toluene and precipitating with ethanol for three cycles and were finally re-dissolved in toluene.

**Synthesis of CIS nanospheres:** The synthetic process was similar to the synthesis of CIS nanoflowers and NPLs, but in this case the injected amount of *t*-DDT was increased still further. Here 2.0 mL of *t*-DDT was injected into the three-neck flask at 180 °C with 0.5 mmol of copper precursor and 0.5 mmol indium precursor in OLA and ODE. Then the reaction was kept at 180 °C for 1 h. The crude CIS nanospheres product solution was dissolved in toluene, and the CIS nanospheres were precipitated by adding ethanol with the aid of centrifugation. All of the CIS nanospheres products were purified by dissolving in toluene and precipitating with ethanol for three cycles, and were finally re-dissolved in toluene.

**Characterization**: Transmission electron microscopy (TEM) was performed using a Philips CM-20 at an accelerating voltage of 200 kV. TEM grids were prepared by depositing one drop of a solution of purified nanoparticles onto an ultrathin carbon-coated grid. High-resolution TEM (HRTEM) was performed using a JEOL 2100F high-resolution transmission electron microscope running at an accelerating voltage of 200 kV with a field-emission gun as an electron source. The grids for HRTEM were prepared by depositing one drop of a solution of purified nanoparticles onto an ultrathin carbon-coated grid. Powder X-ray diffraction (XRD) patterns were obtained using Cu Kα photons from a Bruker D2 Phaser operated at 40 kV and 30 mA. Each sample was deposited as a thin layer on a low background-scattering quartz substrate. Absorption spectroscopy was performed on a Cary 50 ultraviolet–visible spectrophotometer (Varian) using quartz cuvettes. The samples were dissolved in toluene.

## 3. Results and Discussion

**Figure 1:**
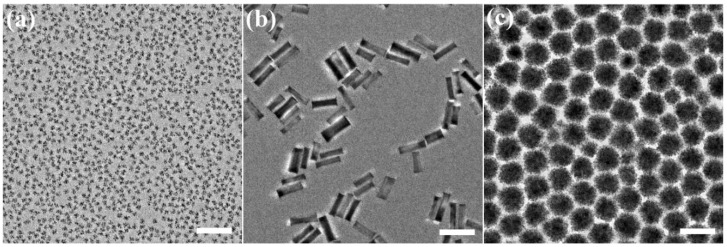
Transmission electron microscopy (TEM) images of CuInS_2_ nanocrystals (CIS NCs) with different shapes, (**a**) nanoflowers, (**b**) nanoplatelets (NPLs), and (**c**) nanospheres. Scale bars are 100 nm for all frames.

Copper iodide and indium acetate were used as metal precursors, which were dissolved in a mixture of 1-octadecene (ODE) and oleylamine (OLA). At 180 °C, the sulfur precursor (*t*-DDT) was injected into the metal precursors solution, and the amount of this reagent largely determined the shape of final CIS NCs, as will be demonstrated below. Figure 1 provides the reaction conditions for CIS NCs with three different shapes, namely nanoflowers, NPLs and nanospheres. CIS nanoflowers were formed when the amount of injected *t*-DDT was 0.5 mL; they had monodisperse sizes of around ~15 nm, and were formed from a few smaller (3–5 nm) CIS nanoparticles (Figure 1a). Similar flower-shaped nanostructures have been widely reported for metal oxides and other semiconductors [21,22,23]. When the amount of injected *t*-DDT was 1.0 mL, the products of the reaction were NPLs with lengths of ~90 nm and widths of ~35 nm (Figure 1b). The amount of the copper and indium precursors had some influence on the width of the CIS NPLs, as shown in Figure S1. When 0.5 mmol of copper iodide and indium acetate were used, the resulting CIS NPLs had the width of ~35 nm (Figure S1a), which changed towards ~30 nm and ~25 nm when those amounts were 1.0 mL (Figure S1b) and 2.0 mmol (Figure S1c), respectively, while the length of the CIS NPLs remained more or less constant at 90 nm.

When the amount of injected *t*-DDT was further increased to 2.0 mL, relatively large (~70 nm in diameter) spherical particles were formed (Figure 1c). Similar to the CIS NPLs, the concentration of the copper and indium precursors and the reaction temperature had an influence on the final size of the CIS nanospheres. When higher concentrations of copper iodide (1.0 mmol) and indium acetate (1.0 mmol) were used in the reaction, the CIS nanospheres formed had a notably smaller size (~50 nm), as shown in Figure S2. At lower reaction temperatures (160 °C), the nanospheres had a smaller size (40–50 nm) with a somewhat broader size distribution than for the CIS nanospheres (70 nm) synthesized at 180 °C (Figure S3b). When the reaction temperature increased to 200 °C, a mixture of CIS NPLs and nanospheres were formed (Figure S3c), and some of the latter appeared to be more of cubic shape. At even higher temperature, the copper and indium precursors decomposed to form metal oxide nanoparticles.

**Figure 2:**
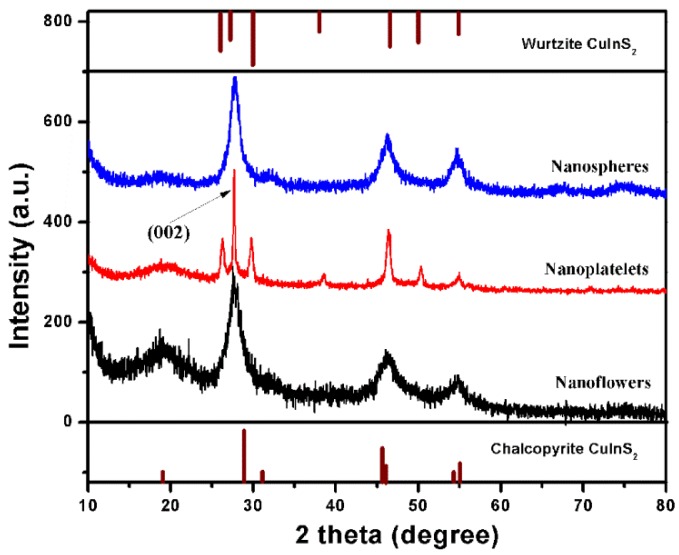
XRD patterns of CIS NCs with different shapes, as indicated on the frame. Standard diffraction peaks from bulk chalcopyrite and wurtzite CIS are given at the bottom and the top, respectively.

Aiming to reveal the structure and the formation mechanism of differently shaped CIS NCs, they were characterized by powder X-ray diffraction (XRD). Figure 2 shows XRD patterns of CIS nanoflowers (black line), NPLs (red line) and nanospheres (blue line). Based on the standard diffraction peaks of bulk CIS with a chalcopyrite and a wurtzite structure given at the bottom and top of Figure 2, respectively, CIS nanoflowers and nanospheres have a cubic chalcopyrite crystal structure, while CIS NPLs have a hexagonal wurtzite crystal structure. The widened diffraction peaks from CIS nanoflowers are consistent with the smaller size of their constituting individual CIS nanoparticles. Sharper diffraction peaks from CIS NPLs indicate the particles’ greater extent in two of three dimensions, as also seen from the TEM image in Figure 1c. The strong Diffraction peak of the (002) plane of wurtzite crystal’s structure (red line in Figure 2) indicates that the growth of the NPLs occurs along the <001> direction; further evidence for this will be discussed below in conjunction with later HRTEM images.

**Figure 3:**
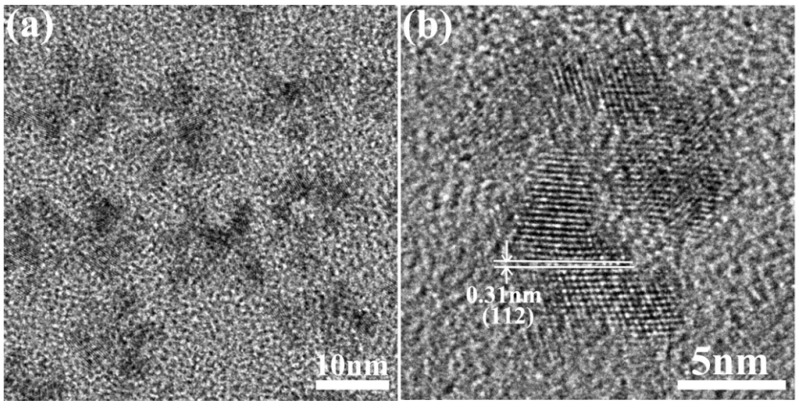
(**a**) TEM image of several CIS nanoflowers, and (**b**) High-resolution TEM (HRTEM) image of an individual CIS nanoflower, showing that they are built by the aggregation of a few CIS NCs.

In order to further reveal the detailed structure of CIS NCs, HRTEM was employed. Figure 3 gives the high-resolution TEM image of several CIS nanoflowers, and a HRTEM image of one individual CIS nanoflower, demonstrating that they were built by the aggregation of a few CIS NCs (typically less than ten particles). The overall size of the CIS nanoflowers is about ~15.0 nm, and that of the constituent CIS NCs is between 3.0~5.0 nm. Smaller size of the constituting CIS NCs in CIS nanoflowers results in broader diffraction peaks in the corresponding XRD pattern (Figure 2). CIS NCs in the nanoflowers are single crystalline, with the lattice planes observed clearly in the HRTEM image in Figure 3b. The 0.31 nm fringe spacing corresponds to the (112) plane in the CIS chalcopyrite crystal structure, which agrees with the crystal structure determined from the XRD pattern in Figure 2 (black trace).

**Figure 4:**
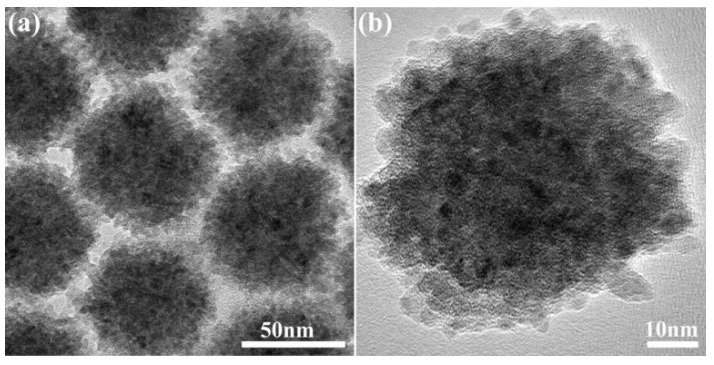
(**a**) TEM image of a close-packed array of CIS nanospheres and (**b**) HRTEM image of an individual CIS nanosphere which is formed by the aggregation of small CIS NCs.

Similar to CIS nanoflowers, 3D CIS nanospheres with an average size of around 70 nm were formed by the aggregation of small CIS NCs, as shown in Figure 4a. Figure 4b gives the HRTEM image of single CIS nanosphere, where lattice fringes can be recognized in the small constituent CIS NCs, aggregated to form a net polycrystalline structure (no overall preferred orientation of the individual particles). A much higher resolution HRTEM image of an edge region of a nanosphere is shown in Figure S4; the 0.31 nm lattice fringe spacing is seen in the individual small CIS NCs, near the periphery corresponding to the distance of the (112) plane in the chalcopyrite crystal structure, in agreement with the crystal structure of CIS nanospheres derived from the XRD pattern in Figure 2 (blue trace).

**Figure 5:**
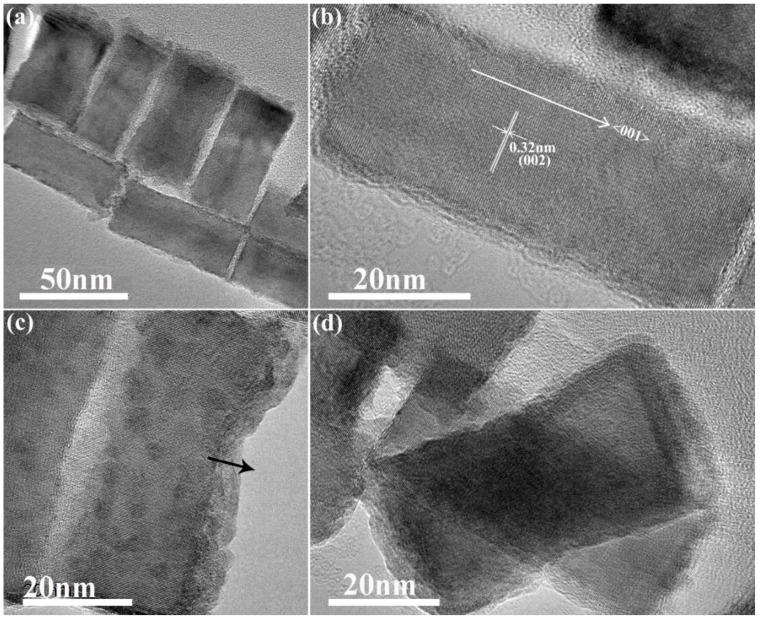
(**a**) TEM image of a few CIS NPLs; (**b**) HRTEM image of a single CIS NPL, demonstrating that its length has been extended along the <001> direction, corresponding to the c-axis of the wurtzite structure; (**c**) HRTEM image of a single CIS NPL with a roughened edge, demonstrating few constituting monolayers; (**d**) HRTEM image of two overlapping CIS NPLs.

NPLs are an interesting class of 2D nanostructures, which can show a strong quantum size confinement effect in one direction (normal to the plate plane). CdSe NPLs with different numbers of layers have been extensively studied, and have showed strikingly narrow absorption and fluorescence spectra [5,7]. For the copper-based chalcogenides, some 2D nanostructures have also been reported, such as CIS hexagonal nanosheets synthesized via a cation exchange method or produced by so-called bottom-up synthesis methods [24,25]. These previously reported CIS nanosheets had wurtzite crystal structures, and the thickness of the NPLs extended along the <001> direction, which is the c-axis in the wurtzite structure. Figure 5a shows a TEM image of the CIS NPLs obtained with our approach, which have rather uniform sizes of around 35 × 90 nm. In the HRTEM image in Figure 5b, the lattice fringes of CIS NPLs are clearly observed, with the 0.32 nm spacing matching that of the (002) plane in CIS with a wurtzite crystal structure, similar to the previously reported cases. However, unlike these examples, our CIS NPLs had the platelet length (rather than the thickness) extended along the <001> direction, corresponding to the c-axis of the wurtzite structure, as shown in Figure 5b. This feature also accounts for the strong diffraction peak of the (002) plane of the wurtzite crystal pattern seen in the XRD pattern (Figure 2, red trace). The thickness of the CIS NPLs is therefore along the <100> direction, which is the a-axis of the wurtzite crystal structure; this is different to the orientation in the previously reported CIS NPLs [24,25].

The thickness is an important parameter for 2D nanostructures, as the quantum size confinement effect can be tuned via the thickness control in such materials. In some CIS NPLs with roughened edges (Figure 5a), several monolayers can be observed (Figure 5c), which translates into a thickness of a few nanometers. Figure 5d displays an image of two overlapping CIS NPLs, which also helps to illustrate how thin they are. The NPL underneath can be still observed clearly, indicating the thickness of the CIS NPL on the top is a few nanometers, because the limited transmission distance of electrons in TEM would otherwise lead to the plate on top fully obscuring the lower-lying NPL.

Figure S5 provides UV-vis absorption spectra of the CIS nanoflowers, NPLs and nanospheres. Nanoflowers have an absorption shoulder around 750~800 nm, which is slightly blue shifted relative to the band edge of the bulk CIS material (around 830 nm) due to the quantum size confinement effect. The small CIS NCs, from which the nanoflowers are formed, have sizes of 3.0~5.0 nm, as shown in Figure 3, and since this range is comparable to the Bohr radius of CIS, a blue shift of absorption occurs. CIS nanospheres show a less distinct shoulder in their absorption spectrum around 750~800 nm, which implies a broader size distribution for the CIS NCs of which they are composed, which is in a good agreement with their TEM image in Figure 4. Similarly, the absorption spectrum of CIS NPLs shows an absorption shoulder around ~750 nm, meaning the quantum size confinement effect is also at play to a similar degree, due to confinement in the thickness direction. As previously reported for Cd-based 2D NPLs, these nanostructures possess thickness dependent optical properties, and also polarized fluorescence emission [5,8,9]. In this work so far, only one particular thickness of CIS NPLs was produced, and their fluorescence emission proved difficult to detect. The future work will focus on thickness-controlled synthesis and appropriate surface passivation for improving the fluorescence of CIS NPLs.

In the preceding syntheses, the shape of CIS NCs could be tuned from nanoflowers to NPLs to nanospheres via changes in the injected amount of *t*-DDT. The *t*-DDT plays a key role in the shape-controlled synthesis of CIS NCs, acting as both a sulfur precursor and a ligand. This reaction component has been used before to synthesize Cu-In-Zn-S nanoflowers [26]; the authors claimed that the decomposition of surface-bound *t*-DDT was the reason for the aggregation of smaller NCs to form larger scale nanoflowers. They also observed that the use of the less *t*-DDT led to the formation of larger size Cu-In-Zn-S nanoflowers. Thus, in order to further understand the role of *t*-DDT in the shape-controlled synthesis of our CIS NCs, some additional ligand-controlled experiments were conducted, using mixtures of 1-DDT and *t*-DDT. Notably, 1-DDT has a similar, but not branched, structure to *t*-DDT, and has also been widely used to synthesize dot-shaped CIS NCs, again fulfilling the roles of both a sulfur precursor and a ligand [13,17]. The size and shape of the produced CIS NCs is shown in Figure S6. When the mixture of 1-DDT (1.0 mL) and *t*-DDT (1.0 mL) was injected into the copper and indium precursor solution instead of 2.0 mL of *t*-DDT only, the resulting CIS NCs showed dot-shaped habits with sizes of 3.0~4.0 nm (Figure S6a). It was observed that 1-DDT dominated the synthesis of these CIS NCs, and the produced nanoparticles showed similar sizes and shapes to those that would be obtained with 1-DDT only. When the injected amount of 1-DDT was decreased to 0.5 mL, and the total injected solution of thiols was kept at a volume of 2.0 mL, the diameter of the synthesized dot-shaped CIS NCs reached ~6.0 nm (Figure S6b). Because of the decreased 1-DDT content, the *t*-DDT started to play a more significant role as a ligand, and larger CIS NCs were obtained. At these synthetic conditions stage, however, 1-DDT still controlled the shape of the CIS NC product. When the injected amount of 1-DDT was further decreased, still proportionately more *t*-DDT could actively participate in the growth and now a transition to more complicated CIS NC shapes was observed (Figure S6c,d). As shown in Figure S6d, where only a little 1-DDT was injected, the latter was not enough to serve as a ligand to all of the CIS NCs. Most of the ligands interacting with the CIS NCs were the now dominant *t*-DDT, so that flower-shaped CIS NCs with a dot-shaped central part were formed (Figure S6d). The formation of such nanostructures may imply that the *t*-DDT ligand primarily induced the aggregation of the CIS NCs to form flower-shaped CIS nanoparticles. Figure S6c illustrates the reaction products formed at an intermediate state; the synthesized CIS NCs have both dot-shapes and flower-shapes. We conclude that the proportion of 1-DDT can be used to induce the formation of dot-shaped CIS NCs, while the presence of *t*-DDT predominantly drives the formation of flower-shaped CIS NCs assemblies. Compared with OLA, such thiol ligands have stronger binding to CIS NC, and thus dominate final shape of the reaction products.

In the same vein as the discussions above, moderating the amount of the *t*-DDT ligand from 0.5 mL to 2.0 mL (as was done in the main part of the discussion above) when used alone can also induce the aggregation of CIS NCs to form differently shaped nanostructures. When 0.5 mL of *t*-DDT was injected into the reaction, it is estimated that ~0.24 mL of the *t*-DDT acted as a sulfur precursor and the remaining ~0.26 mL of *t*-DDT acted as a ligand. The 0.26 mL of *t*-DDT was not enough to cover all of the surface of the CIS NC product, so this role was partially met by the relatively weaker binding OLA which also acted as a ligand for the CIS NCs. The surface of the CIS NCs NCs and some parts of the *t*-DDT ligand coverage could now aggregate at a high temperature, and as a result CIS nanoflowers could then be formed by NC aggregation via the openings provided by the partial *t*-DDT binding over a proportion of the surface substituent NCs. The presence of OLA as a co-ligand prevents the formation of larger CIS NCs, such as nanospheres, and only small-sized CIS nanoflowers were obtained. However, when 2.0 mL of *t*-DDT was injected into the reaction vessel, its overall amount was sufficient to fully meet the role of ligand for the CIS NCs. In this case, all of the produced CIS NCs were sufficiently covered with the *t*-DDT ligand. Related to our discussion of Figure S6, *t*-DDT is a stronger ligand than OLA, and at high temperatures CIS NCs could aggregate in a more extensive manner to form larger nanospheres dominated by this particular ligand. The CIS NPLs (synthesized upon injection of 1.0 mL of *t*-DDT) are the intermediate case between the nanoflowers and nanospheres, where the CIS NCs carry on *t*-DDT as the main ligand and a lesser proportion of the surface is still occupied by OLA. The *t*-DDT ligand then binds preferentially at a high energy surface and the OLA on a lower energy surface, leading to a more ordered assembly into NPLs than that which occurs with either an over- or an under-abundance of the thiol ligand.

## 4. Conclusions

In conclusion, CIS nanostructures with flower-, platelet- and sphere-like shapes were synthesized via a colloidal method, by altering the injected amount of *t*-DDT reactant acting as both a sulfur precursor and a ligand. CIS nanoflowers were formed by the aggregation of a few small (3–5 nm) CIS NCs, and possessed the chalcopyrite crystal structure. Similar to nanoflowers, larger monodisperse CIS nanospheres with diameters of around 70 nm were formed via the aggregation of many small chalcopyrite CIS NCs. CIS NPLs with different widths (25–35 nm) and a constant length (90 nm) could be formed via tuning the concentration of the metal precursors. The thickness of the CIS NPLs is a few nanometers and the evidence of this, provided by HRTEM images, agrees with the blue-shifted location for their absorption edge due to a weak quantum size confinement effect. In contrast to the nanoflowers and nanospheres which possessed the chalcopyrite structure, the NPLs exhibited a wurtzite crystal structure. The formation mechanism driving the formation of each of these various shapes and morphologies of NCs was investigated through a series of ligand-controlled experiments using different proportions of 1-DDT/*t*-DDT ligands. The extent of the *t*-DDT ligand coverage on the initially formed CIS NCs can induce the subsequent aggregation of them to form either nanoflowers of nanospheres at high temperatures. This work provides synthetic pathways for CIS nanostructures with size- and shaped-controlled morphologies, and sheds light on the related formation mechanisms. In the future, the size (length, width, and thickness)-controlled synthesis of CIS NPLs and the related optical property studies will be conducted on this interesting heavy-metal free 2D nanostructure.

## Supplementary Materials

The following are available online at https://www.mdpi.com/2079-4991/9/12/1779/s1, Figure S1. TEM images of CIS NPLs synthesized with different amounts of copper iodide and indium acetate precursors: (a) 0.5 mmol, (b) 1.0 mmol, and (c) 2.0 mmol. The scale bar is 200 nm on each frame. Figure S2. TEM images of CIS nanospheres synthesized with different amounts of copper iodide and indium acetate precursors: (a) 0.5 mmol and (b) 1.0 mmol. The scale bar is 200 nm on each frame. Figure S3. TEM images of CIS nanospheres synthesized at different reaction temperatures: (a) 160 °C, (b) 180 °C and (c) 200 °C. The scale bar is 200 nm on each frame. Figure S4. HRTEM image of an edge region of an individual CIS nanosphere. Figure S5. UV-vis absorption spectra of CIS NCs with different shapes. The curves are vertically offset for clarity. Figure S6. TEM images of CIS NCs synthesized using t-DDT/1-DDT mixtures of different volume ratios, namely: (a) 1.0 mL/1.0 mL, (b) 1.5 mL/0.5 mL, (c) 1.75 mL/0.25 mL, and (d) 1.9 mL/0.1 mL. Blue cartoons illustrate predominant shapes of nanoparticles formed in each case. The scale bar is 200 nm on each frame.
