# Supplementary material for: Shape-Controlled Synthesis of Copper Indium Sulfide Nanostructures: Flowers, Platelets and Spheres
Source: Nanomaterials (Basel). 2019 Dec 14;9(12):1779. doi: 10.3390/nano9121779 (PMC6955946; doi:10.3390/nano9121779)
Supplement: Supplementary file 1 [file nanomaterials-09-01779-s001.pdf]

Supporting Information

Article

Shape-Controlled Synthesis of Copper Indium Sulfide Nanostructures: Flowers, Platelets and Spheres

Jiajia Ning, Stephen V. Kershaw and Andrey L. Rogach *

Department of Materials Science and Engineering, and Centre for Functional Photonics (CFP), City University of Hong Kong, 83 Tat Chee Avenue, Kowloon, Hong Kong, China; jiajning@cityu.edu.hk (J.N.); skershaw@cityu.edu.hk (S.V.K.)

* Correspondence: andrey.rogach@cityu.edu.hk

Received: 3 December 2019; Accepted: 13 December 2019; Published: date

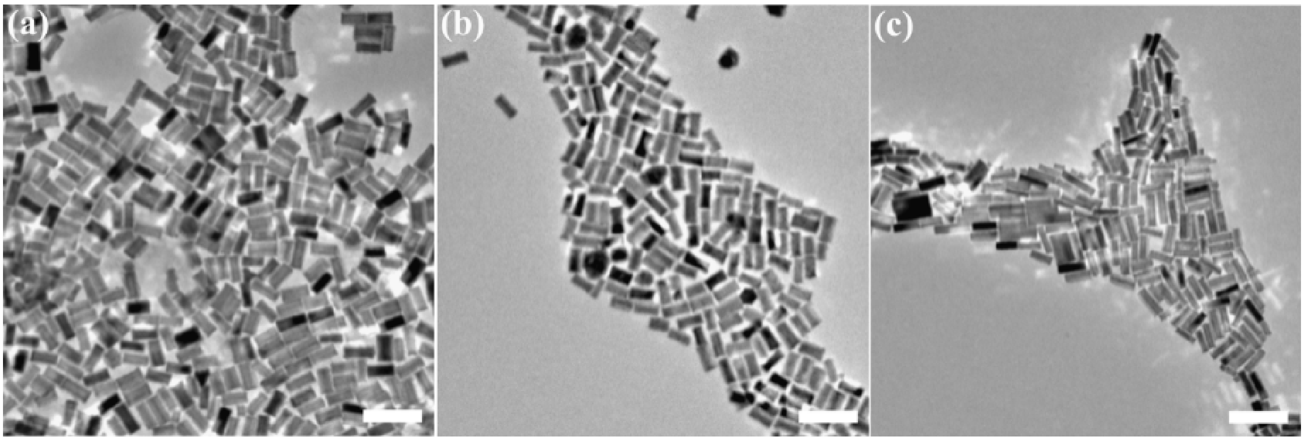

Figure 1. TEM images of CIS NPLs synthesized with different amounts of copper iodide and indium acetate precursors: (a) 0.5mmol, (b) 1.0mmol, and (c) 2.0mmol. The scale bar is 200nm on each frame.

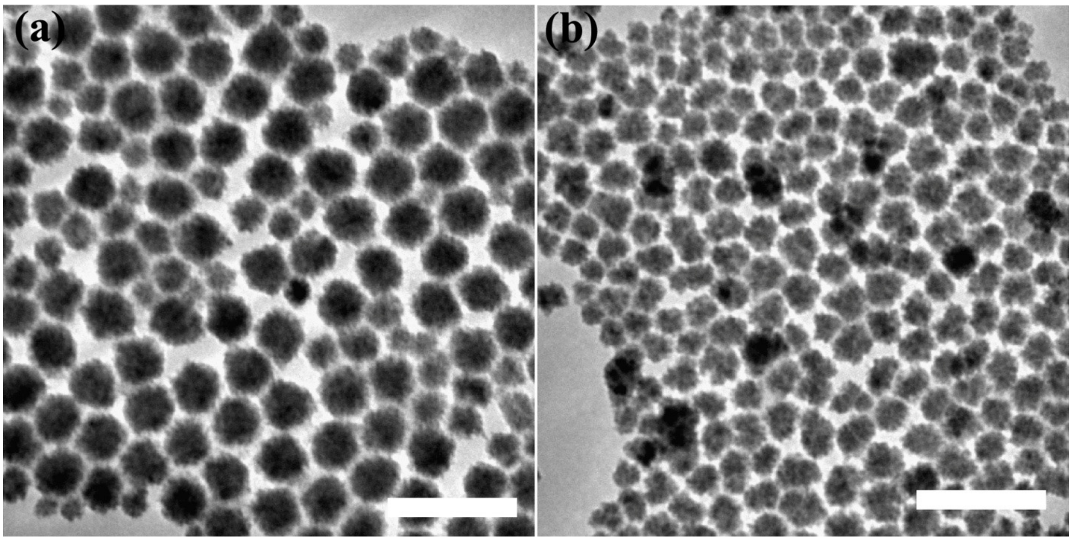

Figure 2. TEM images of CIS nanospheres synthesized with different amounts of copper iodide and indium acetate precursors: (a) 0.5mmol and (b) 1.0mmol. The scale bar is 200nm on each frame.

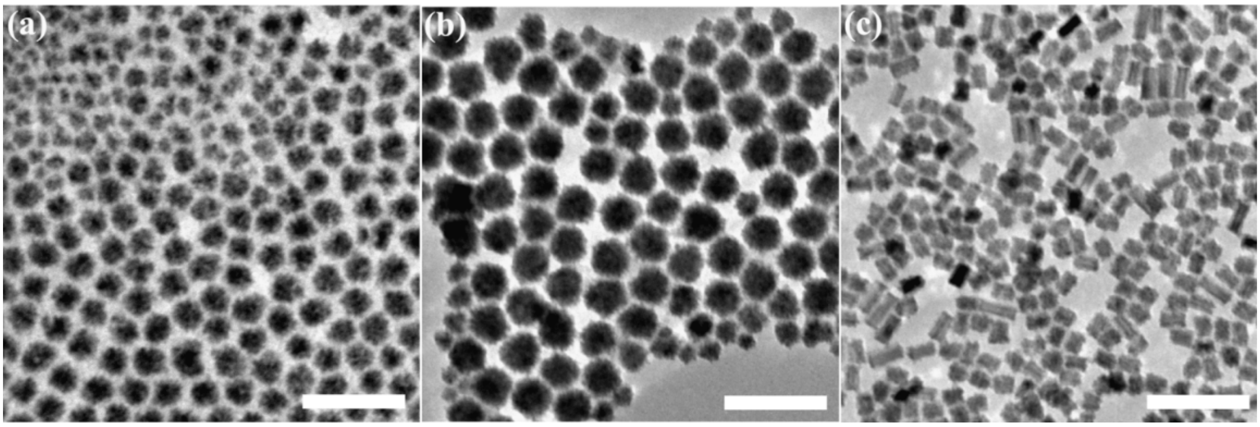

Figure 3. TEM images of CIS nanospheres synthesized at different reaction temperatures: (a) 160°C, (b) 180°C and (c) 200°C. The scale bar is 200nm on each frame.

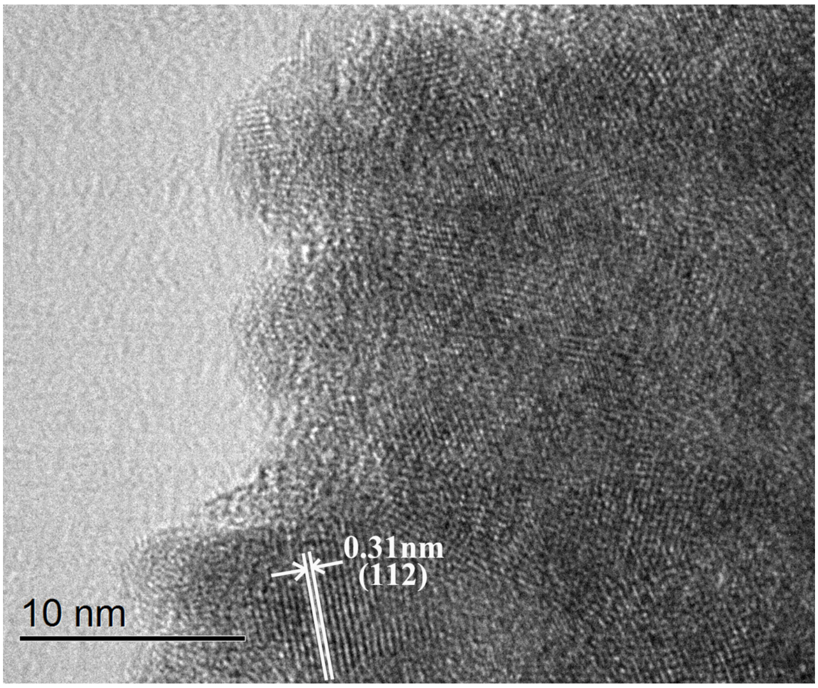

Figure 4. HRTEM image of an edge region of an individual CIS nanosphere.

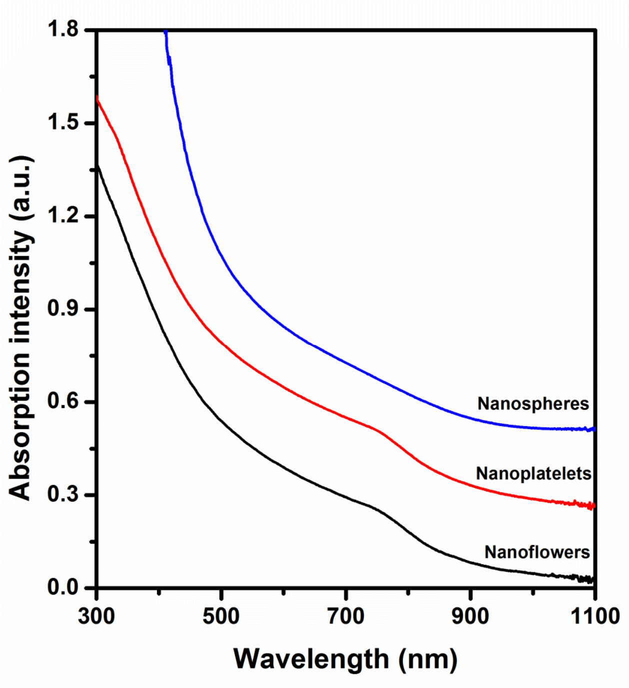

Figure 5. UV-vis absorption spectra of CIS NCs with different shapes. The curves are vertically offset for clarity.

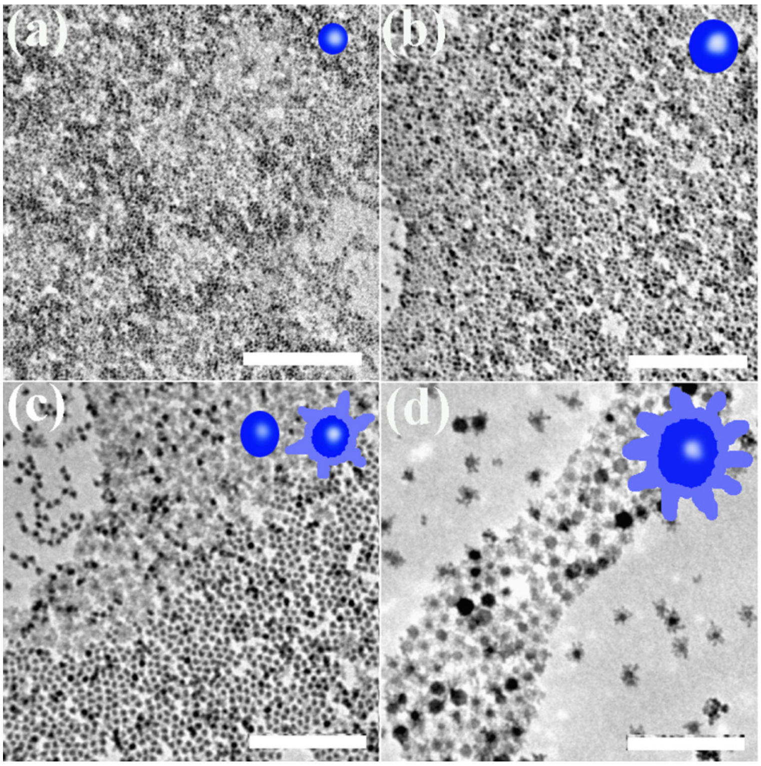

Figure 6. TEM images of CIS NCs synthesized using t-DDT/1-DDT mixtures of different volume ratios, namely: (a) 1.0ml/1.0ml, (b) 1.5ml/0.5ml, (c) 1.75ml/0.25ml, and (d) 1.9ml/0.1ml. Blue cartoons illustrate predominant shapes of nanoparticles formed in each case. The scale bar is 200nm on each frame.
